# Aseptically processed and chemically sterilized BTB allografts for anterior cruciate ligament reconstruction: a prospective randomized study

**DOI:** 10.1007/s00167-012-2309-7

**Published:** 2012-12-01

**Authors:** Peter A. Indelicato, Michael G. Ciccotti, Joel Boyd, Laurence D. Higgins, Benjamin S. Shaffer, C. Thomas Vangsness

**Affiliations:** 1 Department of Orthopaedics and Rehabilitation, University of Florida, 3450 Hull Road, Gainesville, FL 32607 USA; 2 Rothman Institute, Department of Orthopaedic Surgery, Thomas Jefferson University, Philadelphia, PA USA; 3 TRIA Orthopaedic Center, Minneapolis, MN USA; 4 Department of Orthopedic Surgery, Brigham and Women’s Hospital-Ortho, Boston, MA USA; 5 Washington Orthopaedic and Sports Surgery Center, Clinical Orthopedics, George Washington University, Washington, DC USA; 6 Department of Orthopaedic Surgery, University of Southern California, Los Angeles, CA USA

**Keywords:** Anterior cruciate, Allograft, Sterilized, Outcomes, IKDC

## Abstract

**Purpose:**

To compare the clinical outcomes of bone-patellar tendon-bone (BTB) allografts processed via a novel sterilization system with the traditional aseptically processed BTB allografts for anterior cruciate ligament (ACL) reconstruction.

**Methods:**

A total of 67 patients undergoing ACL reconstruction at 6 independent investigation sites were randomized into one of two intervention groups, BioCleanse-sterilized or aseptic BTB allografts. Inclusion criteria included an acute, isolated, unilateral ACL tear, and exclusion criteria included prior ACL injury, multi-ligament reconstruction, and signs of degenerative joint disease. Post-op examiners and patients were blinded to graft type. Patients were evaluated at 6, 12, and 24 months. Clinical outcomes were compared using the IKDC, a KT-1000 knee arthrometer, level of effusion, and ranges of motion (ROM).

**Results:**

After randomization, 24 patients received aseptic BTB allografts and 43 patients received BioCleanse-sterilized allografts. Significant improvement in IKDC scores (*P* < 0.0001) as well as KT-1000 results (*P* < 0.0001) was noted over the 24-month period for both groups. IKDC or KT-1000 results were not significantly different between groups at any time point. Active flexion ROM significantly improved from pre-op to 24-month follow-up (*P* < 0.0001) with no difference between groups at any time point. Active extension ROM did not differ significantly between the two groups.

**Conclusions:**

These results indicate that the sterilization process, BioCleanse, did not demonstrate a statistical difference in clinical outcomes for the BTB allograft at 2 years. The BioCleanse process may provide surgeons with allografts clinically similar to aseptically processed allograft tissue with the benefit of addressing donor-to-recipient disease.

**Level of evidence:**

II.

## Introduction

In anterior cruciate ligament (ACL) reconstruction, the choice of graft has been an area of debate for several years. While there are arguments made that autograft is the gold standard [6, 40], the use of allografts is relatively common with successful outcomes having been well documented in the literature. The use of allograft tissue has been noted to decrease operating time, eliminate donor site morbidity, and increase the tissue available for multi-ligament cases [24, 42]. Clinical studies that evaluated outcomes reported positive results for chronic ligamentous laxity [23]. Subsequently, there have been a number of reports of successful outcomes using allografts in surgery [7, 15, 16]. Further support for the use of allografts can be seen in long-term studies which have reported positive results for patients as long as 10 years after surgery [1, 20, 23]. With the prospect of increased morbidity associated with autograft procedures [2, 12, 43], the rationale for allograft use in ACL surgery seems warranted.

One disadvantage which has received a great deal of attention is that disease can be transmitted as a result of the allograft. Use of allograft tissue has historically been associated with HIV and HCV transmission along with bacterial infections, resulting in significant morbidity and mortality [5]. These risks can be minimized by rigorous donor screening, aseptic harvesting techniques, and tissue processing, but the allograft that is only aseptically processed cannot be guaranteed to be free of all viruses or bacterial spores.

Historically, multiple sterilization techniques have been used for allograft tissue, most notably gamma irradiation and ethylene oxide. The use of gamma irradiation and ethylene oxide has decreased over time in favour of less destructive and biologically friendly chemical cleaning processes [18, 26, 27, 30, 36, 38, 39, 42].

The BioCleanse^®^ tissue sterilization process (RTI Biologics, Alachua, FL) is a non-thermal combination of mechanical and chemical processes that has been reported to inactivate or remove all sources of infectious disease transmission while not compromising the biomechanical and physiological properties of allograft bone and soft tissue [21, 22, 29, 34]. To date, there has not been a direct comparison of clinical outcomes via a randomized, prospective clinical trial with this type of sterilized allograft.

The purpose of this study was to compare the clinical outcomes of patients who underwent ACL reconstruction with BioCleanse BTB allografts to those who received the traditional aseptically, non-irradiated processed BTB allografts. It was hypothesized that patients undergoing ACL reconstruction with BioCleanse BTB allografts would not have statistically or clinically meaningful differences in outcomes from patients who received non-irradiated aseptically processed BTB allografts.

## Materials and methods

Patients who presented with an acute (<4 months), isolated ACL rupture were asked to participate in this study. The patients were at one of 6 independent research sites. A total of 67 patients were randomly assigned, to 1 of 2 groups using the ranblock.exe application, and received either the BioCleanse or aseptic BTB allograft. The mean age of all patients was 34 (SD 9) years. Patients requiring multi-ligament reconstruction, moderate to severe concomitant meniscal repair or ACL revision surgery were excluded. This study was approved by the Institutional Review Board (or equivalent) at all the participating sites and all subjects gave informed consent.

Aseptic grafts were obtained from one of 3 tissue banks currently providing BTB allografts in the United States. All donors were screened, and all tissues were harvested and processed according to standards set forth by the American Association of Tissue Banks [3]. Each investigational site chose their own supplier for the aseptic grafts.

The BioCleanse tissue sterilization system uses a combination of mechanical and chemical processes, working in conjunction with each other. The mechanical component applies oscillating positive and negative pressure in the presence of the chemical agents (including detergents and sterilants), which perfuse the tissue. This combination removes blood and lipids and inactivates or removes pathogenic microorganisms. Repeated rinses throughout the process remove debris, and final rinses remove residual chemicals, leaving the tissue biocompatible [42].

All patients underwent single incision arthroscopic ACL reconstruction under general anaesthesia. A tourniquet was used in every case. All articular damage was noted and recorded using the Outer-bridge classification system. In addition, all meniscal damage was identified, and lesions were treated with partial menisectomy. Following this, a guide system was used that placed the tibial tunnel centre at the anatomical centre of the native ACL. The femoral tunnel was drilled just anterior to the over the top position at the ‘2AM/10PM’ isometric single bundle location using the standard transtibial approach. An endoscopic femoral aimer was used to minimize patient to patient variability. The grafts were reconstituted using room temperature saline for a minimum of 10 min. The BioCleanse grafts were preshaped with a 10 mm diameter and 25–30 mm length bone blocks. The aseptic grafts were shaped individually in each centre during surgery to the same dimensions as the BioCleanse preshaped grafts. The graft was then pulled distal to proximal via the tibial tunnels. Both femoral and tibial fixations were achieved using metallic interference screws. After fixation at the femur, the graft was tensioned with approximately 20 lbs of force for tibial fixation at near extension. Following this, the knee was put through a full range of motion to verify that there was no graft impingement. Stability was then checked to make sure that both the anterior drawer sign and the pivot shift were eliminated. At this point, the arthroscopic instruments were removed and the distal tibial incision was closed in a standard fashion.

All patients followed a uniform ACL rehabilitation protocol. Following surgery, the patients began a therapist-directed physical therapy programme. Post-operative rehabilitation programmes emphasized range of motion restoration, quadriceps strengthening, and patellofemoral joint protection. These were divided into 6 phases using objective criteria for advancement to the next phase; these phases took up to 6 months to complete.

At 3, 6, 12, and 24 months post-operatively, the patients returned to their respective clinics for an evaluation including the International Knee Documentation Committee (IKDC) form along with a physical examination for range of motion (ROM) effusion and a KT-1000 knee arthrometer test. All sites received a training DVD demonstrating KT-1000 testing to ensure standard and consistent use when evaluating the ACL at maximum manual testing. Previous research has shown KT-1000 testing to be reliable and repeatable [14, 25, 28]. Both the examiner and the patient were blinded as to the type of graft implanted.

### Statistical analysis

The IKDC scores, KT-1000 measures, and ROM were compared using a repeated-measures ANOVA. A chi-square test and Fisher’s exact test were used to compare between categorical variables, that is, gender distribution and grade of effusion. Demographic characteristics were compared using a Student’s *t* test. An a priori power analysis was primarily based on KT-1000 measures. Using G3 software (Heinrich Heine University Düsseldorf, Germany) with an effect size of 0.33, alpha of 0.05, power of 0.80, for 3 groups (aseptic, gamma-irradiated, and BioCleanse grafts), we estimated that 112 patients would be required. Due to circumstances such as surgeon preference and patient recruitment, the gamma-irradiated BTB allograft group was dropped. This left the aseptic and BioCleanse grafts for the final analysis. The initial statistical plan had been an unbalanced design, allowing for an unequal number of patients in each group. The level of significance for all statistical tests was set at an alpha level of 0.05.

## Results

As shown in Table 1, there was no significant difference between the groups for age, body mass index, or gender distribution. Of the 43 patients enrolled in the BioCleanse group, there were 29, 24, 20, and 18 tested at 3, 6, 12, and 24 months, respectively. For the aseptic group, testing was completed on 23, 20, 13, and 10 patients at 3, 6, 12, and 24 months, respectively.

**Table 1:** Descriptive data for the patients in this study

	Aseptic allografts	Sterilized allografts	*P* value
Age (years)	31.3 ± 9.2	35.6 ± 8.9	ns
Body mass index	26.7 ± 4.8	26.8 ± 4.7	ns
Revision surgeries	1	1	ns
Male (*n*)	16	25	ns
Female (*n*)	8	18	

**Fig. 1:**
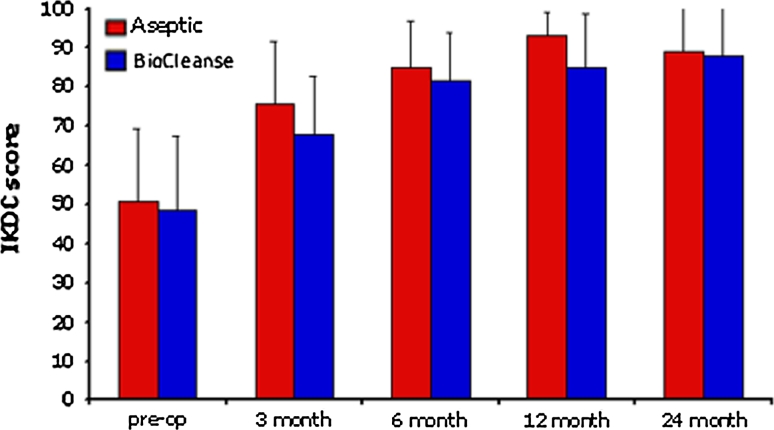
The IKDC scores for the 2 groups. There were no statistically significant differences between the 2 groups at any time point

The data for IKDC scores are presented in Fig. 1. The mean IKDC pre-operatively was 51 (95 % CI 42.3–53.8) and 48 (95 % CI 42.3–53.8) for the aseptic and BioCleanse groups, respectively. This improved over the 24 months to 89 (95 % CI 81.2–96.4) for the aseptic group and 88 (95 % CI 80.6–95.4) for the BioCleanse group. There was no statistically significant difference in IKDC scores at any time point measured.

**Fig. 2:**
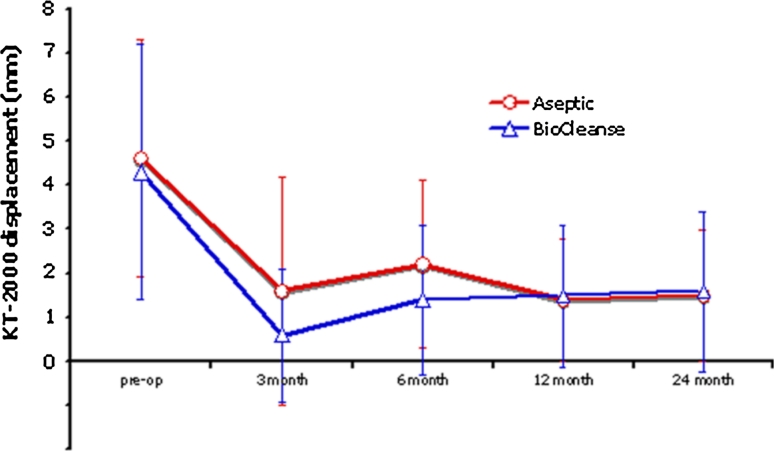
The mean KT-1000 side-to-side differences for anterior displacement at four time points. No significant differences were found between groups

Similarly, the KT-1000 measurements steadily improved over time, from a mean of 4.6 mm (95 % CI 3.7–6.1) and 4.3 mm (95 % CI 3.3–5.3) for the aseptic and BioCleanse groups, respectively. At the end of the study, the anterior knee displacement as measured by the KT-1000 was recorded at 1.6 mm (95 % CI 1.1–2.1) and 1.5 mm (95 % CI 0.9–2.1) for aseptic and BioCleanse groups, respectively (Fig. 2). No statistically significant difference in KT-1000 scores was found between the groups at any time point.

**Fig. 3:**
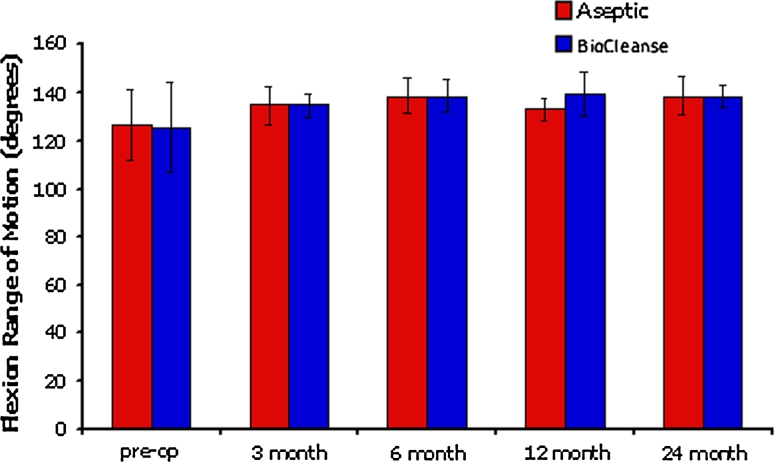
Mean active flexion range of motion for the treated knee for both groups

The results for range of motion (ROM) showed no significant differences between groups at any time point. This was true for the active flexion ROM (Fig. 3) as well as the deficit for passive extension, measured between ipsilateral and contralateral legs. Effusion grading showed no significant differences between the groups. There were no complications noted at 24 months.

There were no cases of either disease transmission or infection that could be attributed to the grafts. This was true for both aseptic and BioCleanse grafts.

## Discussion

This prospectively evaluated clinical outcomes study compared aseptically processed allografts to those treated through the BioCleanse sterilization process. While there have been reports comparing outcomes of irradiated allografts to autografts as well as aseptic allografts, the functional outcomes using BTB grafts that have been chemically, non-thermally sterilized have not been reported in the literature. It should be noted that ‘aseptic’ processing does not necessarily mean that the tissue is free of viruses, bacteria, and/or spores. The possibility of contamination coming from the donor cannot be eliminated and could be inherent to the graft [4], which is why tissue banks attempt to clean with different chemical processes.

A number of clinical studies have been published [13, 15, 19, 22, 32, 35, 37, 38] which compared allografts and autografts in ACL reconstruction. A recent meta-analysis demonstrated no clinical difference when Level I studies were analysed [11]. This study attempted a standardized single bundle, isocentric, anatomical footprint ACL reconstruction for each surgical site to try and ensure consistency within the study.

These results show comparable outcomes between aseptically processed allografts and those that have been sterilized using the BioCleanse process. The importance of this lies in the reduction in the risk of disease transmission which has been documented [5]. Although appropriate donor screening and aseptic harvesting techniques can reduce the risk [17, 33], a method of sterilization that does not damage the structural integrity of the graft is essential. The BioCleanse process inactivates or removes sources of infectious disease transmission without compromising structural integrity of allograft bone and soft tissue [29, 34]. Validation tests of the process have indicated a sterility assurance level of 10^−6^ [18]. The FDA also states that a sterility assurance level of 10^–6^ is necessary for all devices unless there is substantial justification why this level cannot be achieved [43]. An important difference between BioCleanse and other sterilization processes is that BioCleanse does not use irradiation, ethylene oxide, or excessive heat, all of which may adversely impact the properties of the graft tissue [8–10, 26, 36, 41].

There are some limitations to this study. While it may be pointed out that the number of sites may affect reliability, previous literature has pointed to the high intertester reliability when using the KT-1000 [25], and that examiner experience can improve the precision and reliability when using multiple sites [31]. Considering the years of experience of the principal investigators, we expect that the reliability between sites would be high. Statistically, an improved power would make the results more certain. Although 67 patients were initially enrolled in this study, the loss to follow-up resulted in only 18 patients in the BioCleanse group and 10 in the aseptic group at 24 months, which reduced the statistical power in the study. The difference in group sizes had been anticipated in the initial statistical plan for an unbalanced design, yet the diminishing numbers did impact power. It should be noted that the loss of patients to follow-up did affect the power of the final statistical analysis, with the power of the KT analysis calculated to be 0.75, while the power of the IKDC test was calculated to be 0.52.

Also, although the same surgical technique and rehabilitation protocol were applied for all patients at all sites, the role of individual technique as well as adherence to rehabilitation guidelines can introduce variance in the results.

Lastly, while we had initially attempted to compare BioCleanse, aseptic, and gamma-irradiated allografts, extremely low enrolment in the gamma-irradiated arm prevented an adequate statistical comparison. This was due to a general shift away from irradiated allografts and was coupled with several patients who declined to participate in this study when they were informed they might receive an irradiated graft.

## Conclusions

All patients, whether receiving BioCleanse BTB allografts or aseptically processed BTB allografts, exhibited similar clinical outcomes. There were no complications, with only one revision required in each group. This short-term data shows that the BTB allografts processed through the BioCleanse process provide a viable option for ACL reconstruction while minimizing the risk of disease transmission.
